# Absence of the Klotho Function Causes Cornea Degeneration with Specific Features Resembling Fuchs Endothelial Corneal Dystrophy and Bullous Keratopathy

**DOI:** 10.3390/biology13030133

**Published:** 2024-02-20

**Authors:** Chun-Yen Wu, Da-Fong Song, Zhi-Jia Chen, Chao-Sheng Hu, David Pei-Cheng Lin, Han-Hsin Chang

**Affiliations:** 1 Department of Nutrition, Chung Shan Medical University, Taichung City 402, Taiwan; jefferywuu1992@gmail.com (C.-Y.W.); a0918875372@gmail.com (D.-F.S.); leo.cmu.ssm.110601003@gmail.com (C.-S.H.); 2 Department of Medical Laboratory and Biotechnology, Chung Shan Medical University, Taichung City 402, Taiwan; j5657849044@gmail.com; 3 Department of Ophthalmology, Chung Shan Medical University Hospital, Taichung City 402, Taiwan

**Keywords:** Klotho null mutation, age-related cornea degeneration, Fuchs endothelial corneal dystrophy, bullous keratopathy, dry eye disease, murine model

## Abstract

**Simple Summary:**

The Klotho null mutation leads to accelerated senescence in many organs. The effects of an absence of the Klotho function on the cornea remain largely unknown. This study elucidates the impact of the Klotho loss-of-function on corneas. The results show endothelial cell-shedding, reduced corneal epithelial cell density, and decreased cornea stromal layer thickness in Klotho mutant mice. Furthermore, guttae formation and the desquamation of wing cells were significantly increased with the Klotho loss of function, comparable to the conditions of Fuchs endothelial corneal dystrophy and bullous keratopathy. The mechanism analysis showed oxidative stress-induced apoptosis, inflammation, and extracellular matrix remodeling, resulting in the disruption of epithelial repair and maintenance. Thus, the Klotho null mutation model can be useful for studying cornea degeneration, encompassing senescent corneal diseases, such as dry eye, Fuchs endothelial corneal dystrophy, and bullous keratopathy.

**Abstract:**

The Klotho loss-of-function mutation is known to cause accelerated senescence in many organs, but its effects on the cornea have not been published. The present study aims to investigate the effects of the Klotho null mutation on cornea degeneration and to characterize the pathological features. Mouse corneas of Klotho homozygous, heterozygous, and wild-type mice at 8 weeks of age for both genders were subject to pathological and immunohistological examinations. The results show an irregular topography on the corneal surface with a Klotho null mutation. Histological examinations revealed a reduced corneal epithelial cell density, endothelial cell-shedding, and decreased cornea stromal layer thickness in the absence of the Klotho function. Furthermore, guttae formation and the desquamation of wing cells were significantly increased, which was comparable to the characteristics of Fuchs endothelial corneal dystrophy and bullous keratopathy. The mechanism analysis showed multi-fold abnormalities, including oxidative stress-induced cornea epithelium apoptosis and inflammation, extracellular matrix remodeling in the stroma, and a disruption of epithelial repair, presumably through the epithelial–mesenchymal transition. In conclusion, cornea degeneration was observed in the Klotho loss-of-function mutant mice. These pathological features support the use of Klotho mutant mice for investigating age-related cornea anomalies, including Fuchs endothelial corneal dystrophy, bullous keratopathy, and dry eye diseases.

## 1. Introduction

The cornea is on the exterior surface of the eye and is crucial for vision due to its role as an optic medium for the visual axis and maintenance of transparency. The structure of the cornea gradually increases in thickness from the central cornea to the periphery in a convex, aspheric shape, providing a proper anterior reflective surface for the eye. Due to its transparent and avascular design, the cornea transmits incoming light to focus onto the retina with minimum scattering and optical degradation [1,2,3].

Senescence is a multifactorial process that occurs due to an accumulation of biological and pathogenic changes. Age is an important factor in causing ocular diseases, including corneal degenerations [4,5]. Over time, aging leads to a gradual deterioration of the cornea’s structure, composition, and physiological functions [6], eventually affecting its ability to repair itself and protect itself against potential damage from the outer environment [5]. Senescent corneal cells are generally transformed into a highly catabolic phenotype [5], during which the activities of collagenase and MMPs are increased along with their degradation ability [7], leading to an age-associated decrease in total corneal thickness [1]. Also, notably, a decrease in keratocyte density occurs during aging [5]. Since collagen fibers are derived from keratocytes, a decrease in keratocyte density aggravates the collagen loss in the cornea stroma, contributing to the reduction in total cornea thickness during aging [5].

Aging also affects corneal endothelial cells. Endothelial cells are distorted and atrophied during aging, along with an increase in Descemet’s membrane thickness [6]. One example of age-related endothelial atrophy is the condition of Fuchs endothelial corneal dystrophy (FECD) [8], a complex age-related genetic disorder [9,10] that begins in the central cornea [11]. Mature corneal endothelial cells (CECs) do not have the ability to regenerate [8], but are liable to be damaged by the elevated oxidative stress associated with aging [2,10], leading to accelerated endothelial cell loss [8], with a subsequent accumulation of excess fluid in the cornea. This scenario results in corneal edema and the loss of corneal transparency [2], and eventually corneal blindness [8]. Another hallmark of FECD is the formation of dome-shaped extracellular deposits called guttae, resulting from the excrescences of collagen from stressed corneal endothelial cells [12]. As the CECs become atrophic, FECD causes corneal edema and progressively develops into bullous keratopathy (BK) [13]. With BK, epithelial exfoliation is significantly increased [14], leading to the formation of epithelial blisters [2] and causing severe pain when the blisters rupture [8].

The clinical manifestations of FECD at early stages are hardly distinguished from the corneal changes during normal aging [11]. Also, due to the complex etiology of FECD, which involves both genetic and environmental factors, there is currently a lack of therapeutic intervention [12]. Thus, the extensive research on its mechanisms are urgently needed and some animal research models have been developed. For example, transcorneal cryoinjury is applied to cause corneal epithelial ruptures or mechanical damage is performed to disrupt Descemet’s membrane (DM) [15] to mimic FECD pathogenesis. Another non-genetic FECD animal model was employed based on the physiological susceptibility to ultraviolet A (UVA)-induced damage, demonstrating that corneal exposure to chronic oxidative stress can cause FECD with late-life manifestations [9]. However, since effective treatments are better administered during early diagnosis and intervention, animal models with an early onset of disease are more favorable for the research and development of therapeutic regimens. Thus, FECD animal models that manifest at early stages, particularly those that can be used to distinguish disease pathogenesis from normal aging, are highly demanded.

The Klotho mutation is known to cause accelerated aging, with a dramatic phenotype of a shortened life span in mice, which can be reversed with a Klotho overexpression [16]. Thus, the Klotho pathway is generally believed to involve chronic disorders related to aging. When the pathway is disrupted, increased oxidative stress occurs, resulting in a wide range of pathological consequences, including ocular diseases in the lens, retina, and optic nerve [17,18,19]. Nevertheless, little attention has been paid to the effects of the Klotho null mutation on the ocular surface.

In this study, we investigate whether Klotho null mice exhibit ocular surface disorders.

## 2. Materials and Methods

### 2.1. Procurement of Klotho Null Mutant Mice

The Klotho null mutant mice with genetic backgrounds on C57BL/6J were purchased from the Mutant Mouse Resource and Research Center (MMRRC), UC Davis, Davis, CA, USA. The mice were initially bred in the National Laboratory Animal Center (NLAC), Taipei City, Taiwan. After several generations, the mice were transferred and maintained in the animal facility at Chung Shan Medical University, Taichung City, Taiwan, and kept under temperatures between 20 and 24 °C with a humidity ranging from 50% to 55%. The mice were maintained under a normal 12 h light/12 h dark cycle. The mice were allowed to take commercial diets and water ad libitum throughout the entire experiment. The protocol was reviewed and approved by the Institutional Animal Care and Use Committee of Chung Shan Medical University (IACUC Approval No: 2472). Throughout the entire study, all experimental procedures were performed following the standard laboratory animal procedures according to the ARVO Statement for the Use of Animals in Ophthalmic and Vision Research.

### 2.2. Genotyping of Klotho Mutant Mice

PCR genomic DNA amplification was performed to determine the mouse genotypes. The PCR was based on a protocol published by the Mutant Mouse Resource and Research Center (MMRRC) at the University of California, Davis. Two primer sets were used, Primer 1 (GATGGGGTCGACGTCA) and Primer 2 (TAAAGGAGGAAAGCCATTGTC), to amplify for 186 bp PCR products, while Primer 3 (GCAGCGCATCGCCTTCTATC) and Primer 4 (ATGCTCCAGACATTCTCAGC) for 455 bp PCR products. The thermocycler parameters were set at 94 °C, 5 min for initiation; 94 °C, 15 s for denaturation; 65 to 55 °C, 15 s for annealing; and 72 °C, 30 s for elongation. A total of 40 cycles of denaturation–annealing–elongation were performed. A final amplification at 72 °C for 5 min was performed before the termination of PCR cycling. The final PCR products were preserved at 4 °C and run for electrophoresis on gels. The obtainment of only 455 bp was regarded as homozygotes, both 186 bp and 455 bp PCR products as heterozygotes, and only 186 bp as wild types. All analyses in this study were conducted on 8-week-old mice of both genders and in all three genotypes.

### 2.3. Cornea Surface Topography

The light reflections swept across the cornea were used for cornea surface topography analysis [20]. Briefly, images of cornea surfaces were taken with a stereoscopic zoom microscope equipped with an above ring illuminator (SMZ 1500; Nikon, Tokyo, Japan). The cornea surface topography scores were calculated based on the digital images. Each cornea surface image covered 5 concentric circles reflecting the light from the illuminator. Each concentric circle was divided into 4 quadrants. A 5-point scale based on the number of distorted concentric circles in each quadrant was applied: 0, no distortion; 1, distortion in a circle; 2, distortion in 2 circles; 3, distortion in 3 circles; 4, distortion in 4 circles; and 5, distortion in all 5 circles [21,22]. A total score was obtained by adding up the points from all 4 quadrants.

### 2.4. Tissue Fixation, Micro-Section, and Hematoxylin–Eosin Staining

Mouse eyes were extracted from the mice after euthanasia and then transferred to the Davidson’s solution (8 mL of 37% formaldehyde, 87 mL of 50% ethanol, and 5 mL of glacial acetic acid in a total preparation of 100 mL) for 18–24 h for fixation, followed by standard embedding procedures in paraffin. The tissues were sectioned at 5 μm in thickness and allowed to air-dry at room temperature. The tissue sections were processed to dewax them and the exposed to water for rehydration. The preparations were then stained with Mayer’s Hematoxylin (Cat. no. S3309, Dako, Carpinteria, CA, USA), followed by washing 3 times and de-staining with acidic alcohol, and then subjected to staining with 4% Eosin Y (Cat. no. E6003, Sigma-Aldrich, St. Louis, MO, USA). After being thoroughly air-dried at room temperature, the preparations were mounted by coverslips with Micromount (Cat. no. 3801731, Leica Biosystems, Deer Park, IL, USA).

### 2.5. Immunohistochemical and Immunofluorescent Detections

Specific antigens were detected using corresponding antibodies with signals detected by either a non-fluorescent or fluorescent system. The slides were processed through de-waxation and rehydration. The preparations were then microwaved for antigen retrieval in a citrate buffer. After cooling down, the slides were treated with 5% H_2_O_2_ for at least 10 min, washed several times in tap water, and soaked in 1% blocking buffer to block non-specific binding. An antibody was prepared in the blocking solution and applied onto the slides for antigen binding. The slides were covered with a coverslip and kept at 4 °C overnight, followed by being immersed in TBS to release the coverslips before detection.

For the non-fluorescent detection, a Super-Sensitive Polymer HRP IHC Detection System (Cat. no. QD400-60KE, Biogenex, Adibatla, Telengana, India) was used according to the manufacturer’s protocol. The preparations were immersed in a super-enhancer solution for 40 min, washed several times with TBS, and covered with HRP conjugates for 2 h. After that, the slides were washed with TBS before the application of DAB (3,3′-Diaminobenzidine) for color detection and counterstaining by Hematoxylin. The antibodies used for the detection were the anti-Klotho antibody (Abcam, Cambridge, UK, cat. no. ab181373, dilution at 1/100), anti-MDA (malondialdehyde) (Abcam, Cambridge, UK, cat. no. ab243066, dilution at 1/500), anti-NALP3 (Arigobio, Hsinchu City, Taiwan, ROC, cat. no. ARG40539, dilution at 1/500), anti-Ki67/MKI67 (Novusbio, Centennial, CO, USA, cat. no. NB110-89719, dilution at 1/500), and anti-Wnt1 (Affbiotech, Jiangsu, China, cat. no. AF5315, dilution at 1/500).

For immunofluorescent detection, the primary antibodies were added to sections and kept at 4 °C in a humid box overnight. The sections were then rinsed with TBS three times and incubated with a secondary antibody: Goat Anti-Rabbit IgG H&L (Alexa Fluor^®^ 488) (Abcam, Cambridge, UK, cat. no. ab150077, dilution at 1/500). The primary antibodies used for the detection were anti-Nrf2 (nuclear factor erythroid 2-related factor 2) (Affbiotech, Jiangsu, China, cat. no. AF0639, dilution at 1/500), anti-8-OHdG (8-hydroxy-2-deoxyguanosine) (GeneTex, Irvine, CA, USA, cat. no. GTX35250, dilution at 1/400), anti-p53 (GeneTex, Irvine, CA, USA, cat. no. GTX102965, dilution at 1/500), anti-MMP2 (GeneTex, Irvine, CA, USA, cat. no. GTX104577, dilution at 1/500), and anti-MMP9 (Abcam, Cambridge, UK, cat. no. ab38898, dilution at 1/500). DAPI (cat. no. D9542; Sigma-Aldrich, St. Louis, MO, USA) staining was performed for 5 min at room temperature to locate nucleoli. The preparations were viewed and photographed under a fluorescent microscope (Axio Imager A2; ZEISS, Oberkochen, Germany) with activation/emission wavelengths of 358/463 nm for DAPI and 495/519 nm for FITC, respectively.

### 2.6. In Situ TUNEL Staining

To detect the apoptosis of corneas, TUNEL staining was performed using a One-Step TUNEL In Situ Apoptosis Kit (Elabscience, Houston, TX, USA, cat. no. E-CK-A325), following the supplier’s protocol. The preparations were then stained with DAPI and covered with coverslips. The fluorescence signals were visualized and photographed using a Carl Zeiss Microscope (Axio Imager A2; ZEISS, Oberkochen, Germany) with activation/emission wavelengths of 358/463 nm for DAPI and 495/519 nm for TUNEL, respectively.

### 2.7. Statistics

The quantification of all staining was allocated according to the central, peripheral, and limbal parts along the cornea (Supplementary Figure S1). The quantification of positive signals was calculated with the ImageJ program accompanied by a Color Deconvolution 2 plugin, free software provided by Gabriel Landini [23], according to a protocol that was published previously [24]. The images were initially processed through a series of procedures, including color deconvolution, addition of vectors, and adjustment of threshold to exclude non-positive areas. After that, manual measurements of the defined regions of interest were performed for the final results. The data were presented as the area of interest divided by the total area of the cornea.

GraphPad Prism v9 was used to perform a two-way ANOVA (or mixed model) for the assessment of the wild types, Klotho heterozygotes, and Klotho homozygotes for both genders; significant differences were denoted as * *p* < 0.05, ** *p* < 0.01, *** *p* < 0.001, and **** *p* < 0.0001, respectively.

## 3. Results

### 3.1. Cornea Surface Irregularity with Klotho Null Mutation

**Figure 1:**
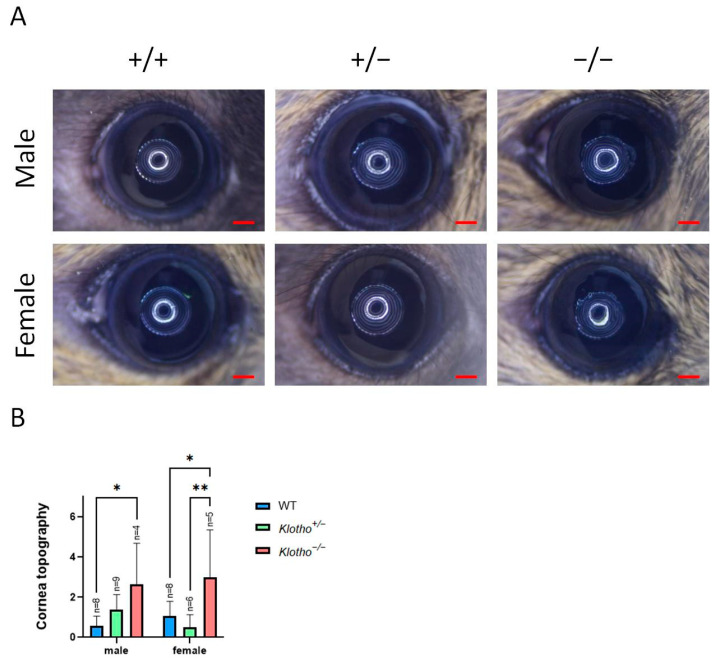
(**A**) The mouse corneal topographies of two genders and three different genotypes are shown by representative photographs. Scale bars represent 200 μm. (**B**) Quantification of corneal surface topography shows significantly increased scores for the Klotho null mutants, with * *p* < 0.05 and ** *p* < 0.01.

Cornea surface topography was used to examine and compare the ocular surface alterations among the three genotypes and for both genders (Figure 1A). A significant increase in topography scores was observed between the male wild-type and the Klotho homozygous corneas (Figure 1B). In the female mice, a significant increase in topography scores was observed between the wild-type and the Klotho homozygous corneas, as well as between the corneas of the heterozygotes and the homozygotes (Figure 1B).

### 3.2. Histological Analysis Indicates Cornea Pathological Alterations with a Klotho Null Mutation

**Figure 2:**
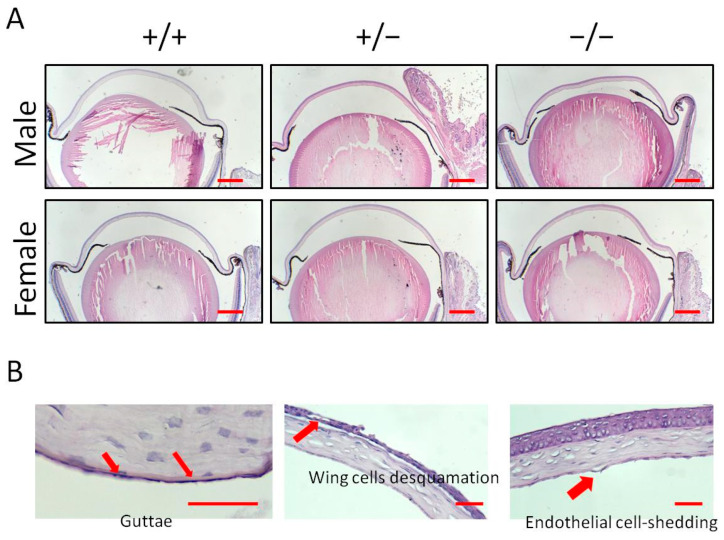
(**A**) Cornea sections of the two genders and the three genotypes stained with Hematoxylin–Eosin shown by representative photographs. Scale bars represent 200 μm. (**B**) Pathological changes occurred with the Klotho null mutation, showing guttae formation, wing cell desquamation, and endothelial cell-shedding (indicated by red arrows). Scale bars represent 25 μm.

To further characterize the differential cornea damage, histological sections with Hematoxylin–Eosin staining were examined (Figure 2A). For the Klotho null mutation, guttae formation, wing cell desquamation, and endothelial cell-shedding were observed (Figure 2B). The quantitative analysis showed 5/5 (100%), 3/5 (60%), and 3/5 (60%) of guttae formation, wing cell desquamation, and endothelial cell-shedding, respectively, in the male Klotho homozygous corneas (Table 1). In the female Klotho homozygous corneas, 3/4 (75%), 2/4 (50%), and 2/4 (50%) of guttae formation, wing cells desquamation, and endothelial cell-shedding were observed, respectively. The percentages of these cornea pathological alterations were higher than those of the wild types and also higher than those of the heterozygous corneas.

**Figure 3:**
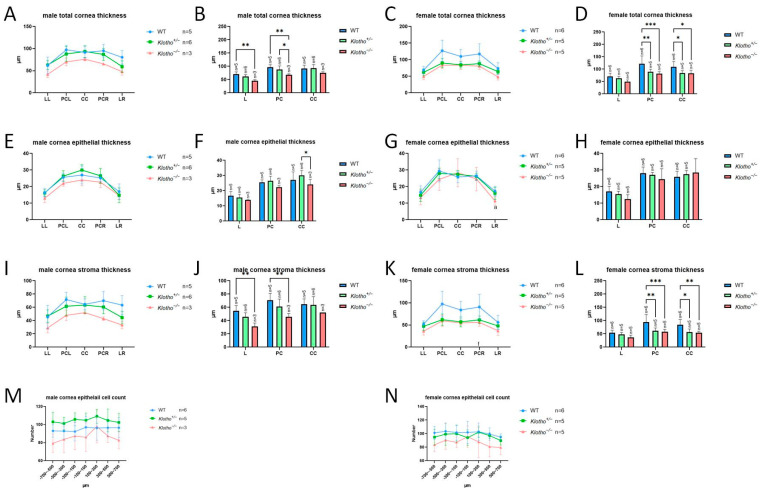
(**A**–**L**) Quantification of total cornea, epithelium, and stroma thickness for central cornea (CC), peripheral cornea (PC), and limbus (L), respectively. Limbus left (LL); limbus right (LR). The total cornea thickness of the homozygous cornea is reduced (**A**–**D**), but no significant thickness reduction can be seen in the cornea epithelial layer (**E**–**H**), and the epithelial cell number shows a decreasing trend, but without significance (**M**,**N**), with * *p* < 0.05, ** *p* < 0.01, and *** *p* < 0.001.

The total cornea thickness of the homozygous cornea was reduced, compared with that of the wild-type and heterozygous corneas (Figure 3A–D). Further analysis showed that the reduction in total cornea thickness was not due to the significant thickness reduction in the cornea epithelial layer (Figure 3E–H) and the epithelial cell number was not reduced (Figure 3M,N). Rather, the reduction was mainly caused by the thinning of the cornea stroma (Figure 3I–L).

### 3.3. Significant Elevations of MMP2, MMP9, and Wnt-1 Expression with the Klotho Null Mutation

**Figure 4:**
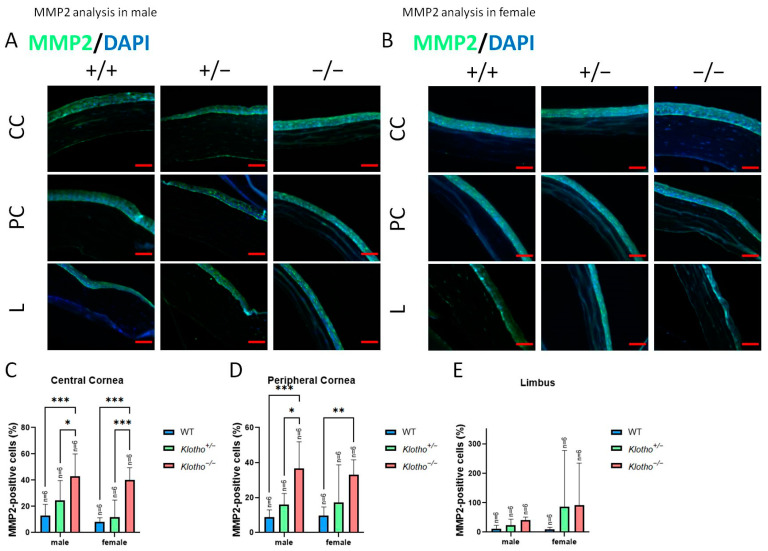
Representative photographs of corneal immunofluorescent detection with MMP2 antibody and quantification analysis. (**A**) MMP2 immunodetection in a male cornea. (**B**) MMP2 immunodetection in a female cornea. (**C**–**E**) To analyze MMP2 quantitatively, presentations of the MMP2-positive area/central cornea area, peripheral cornea area, and limbus area are shown in Figures (**C**–**E**), respectively. MMP2 is significantly increased in the central and peripheral corneas in the Klotho homozygous mutants (**C**,**D**). In the limbus, an increase in MMP2 can also be observed for both genders of the Klotho homozygotes, compared with the wild types and heterozygotes, although without significance (**E**). * *p* < 0.05, ** *p* < 0.01, and *** *p* < 0.001. The scale bars represent 50 μm.

**Figure 5:**
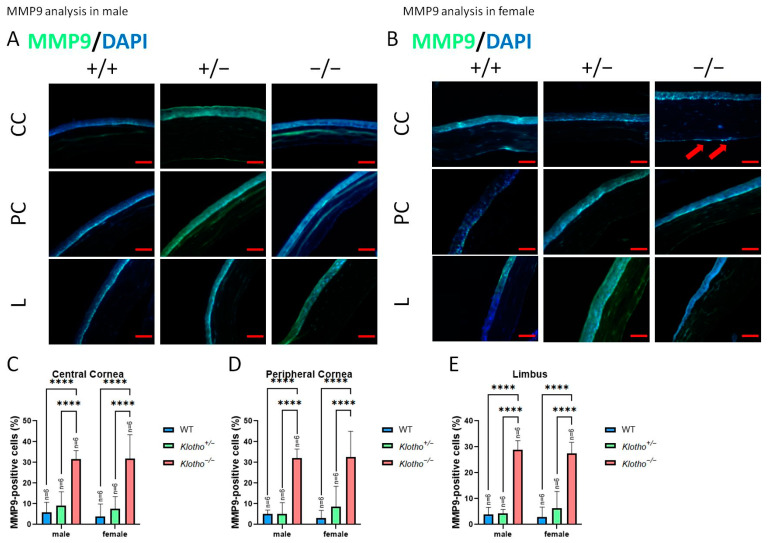
Corneal immunofluorescent detection with MMP9 antibody as shown by representative photographs and quantitative analysis. (**A**) MMP9 immunodetection in a male cornea. (**B**) MMP9 immunodetection in a female cornea. (**C**–**E**) To analyze MMP9 quantitatively, presentations of the MMP9-positive area/central cornea area, peripheral cornea area, and limbus area are shown in Figures (**C**–**E**), respectively. MMP9 is significantly increased in not only in the central and peripheral corneas, but also in the limbus (**A**–**E**). Notably, guttae formation is co-localized with MMP9 expression (**B**, indicated by red arrows). **** *p* < 0.0001. Scale bars represent 50 μm.

Since we observed a decrease in cornea total thickness under the influence of the Klotho null mutation, the expression levels of MMP2 and MMP9 were investigated (Figure 4A,B). The quantitative analysis showed that MMP2 was significantly increased in central and peripheral corneas in the Klotho homozygous mutants (Figure 4C,D, respectively), irrespective of the gender. In the limbus, an increase in MMP2 was also found for both genders of the Klotho homozygotes, compared with the wild types and heterozygotes, although without significance (Figure 4E). With the Klotho null mutation, MMP9 was significantly increased in not only the central and peripheral corneas, but also in the limbus (Figure 5A–E). Notably, the guttae formation was co-localized with MMP9 expression (Figure 5B, indicated by red arrows).

**Figure 6:**
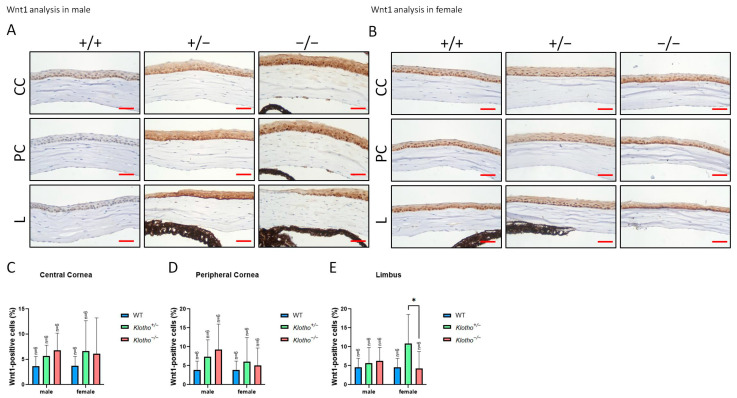
Representative photographs of corneal immunohistochemical detection with Wnt1 antibody and quantification analysis. (**A**) Wnt1 immunodetection in a male cornea. (**B**) Wnt1 immunodetection in a female cornea. (**C**–**E**) For the quantitative analysis, the Wnt1 expression is presented as a Wnt1-positive area/central cornea area, peripheral cornea area, and limbus area in Figures (**C**–**E**), respectively. Wnt-1 expression increases with the Klotho null mutation in both genders, although without statistical significance (**C**,**D**). In the limbus, Wnt-1 expression also increases in the male Klotho null mutants. In the limbus of the female mutants, a significant decrease in Wnt-1 expression can be seen when compared with the Klotho heterozygous mutants (**E**). * *p* < 0.05. Scale bars represent 25 μm.

Wnt-1 expression was also examined in the three genotypes (Figure 6A,B). Quantitatively, in the central and peripheral corneas, Wnt-1 expression was increased with the Klotho null mutation in both genders, although without statistical significance (Figure 6C,D). In the limbus, Wnt-1 expression was also increased in the male Klotho null mutants, whereas Wnt-1 expression was significantly decreased in the female mutants (Figure 6E).

### 3.4. The Status of NLRP3 with the Klotho Null Mutation

**Figure 7:**
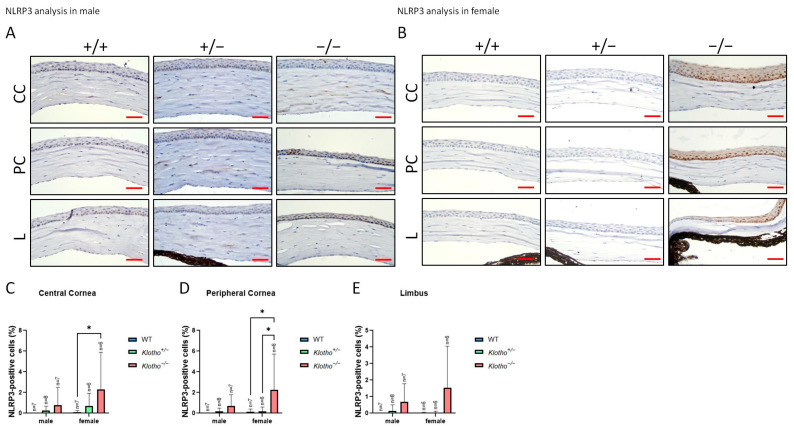
Representative photographs of corneal immunohistochemical detection with NLRP3 antibody and quantification analysis. (**A**) NLRP3 immunodetection in a male cornea. (**B**) NLRP3 immunodetection in a female cornea. (**C**–**E**) To analyze this quantitatively, NLRP3 expression is presented as the NLRP3-positive area/central cornea area, peripheral cornea area, and limbus area. Significant NLRP3 elevations can be seen in the female central and peripheral corneas with Klotho null mutations (**C**,**D**). * *p* < 0.05. Scale bars represent 25 μm.

For the further characterization of the influence of the Klotho loss-of-function mutation on the cornea, we examined the NLRP3 expression. The data show that NLRP3 generally increases with the Klotho null mutation, irrespective of gender (Figure 7A,B). In particular, a statistically significant NLRP3 elevation was found in the female central and peripheral corneas with Klotho null mutations (Figure 7C,D).

### 3.5. Increased Oxidative Stress as Indicated by 8-OHdG and MDA Markers

**Figure 8:**
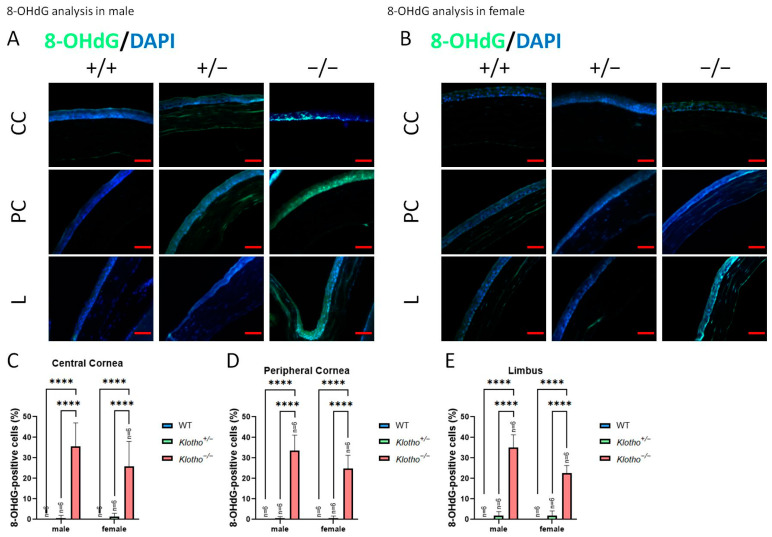
Representative photographs of corneal immunofluorescent detection with 8-OHdG antibody and quantification analysis. (**A**) 8-OHdG immunodetection in a male cornea. (**B**) 8-OHdG immunodetection in a female cornea. (**C**–**E**) To analyze this quantitatively, 8-OHdG expression is represented by the 8-OHdG-positive area/central cornea area, peripheral cornea area, and limbus area. The accumulation of 8-OHdG is found to be highly significant in all three regions of the cornea under the influence of the Klotho null mutation, when compared with those of the wild types and the heterozygotes (**C**–**E**). **** *p* < 0.0001. The scale bars represent 50 μm.

**Figure 9:**
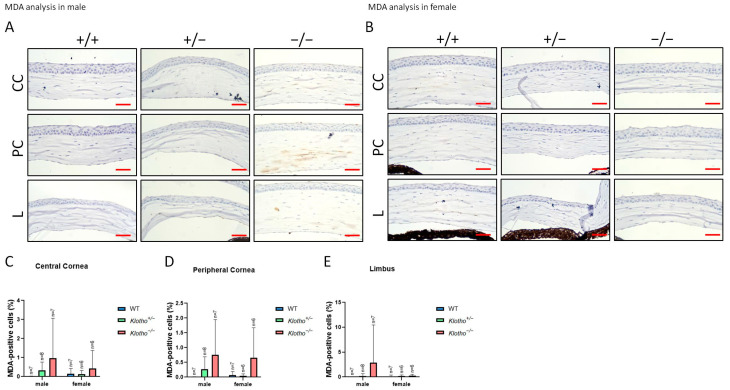
Corneal immunohistochemical detection with MDA antibody as shown by representative photographs and quantitative analysis. (**A**) MDA immunodetection in a male cornea. (**B**) MDA immunodetection in a female cornea. (**C**–**E**) To analyze this quantitatively, MDA expression is represented by the MDA-positive area/central cornea area, peripheral cornea area, and limbus area. The accumulation of MDA also increased, although without statistical significance (**C**–**E**). The scale bars represent 25 μm.

Since NLRP3 was generally increased with the Klotho null mutation, we further examined the oxidative stress status as reflected by 8-OHdG and MDA markers (Figure 8A–E and Figure 9A–E). The accumulation of 8-OHdG was found to be highly significant in all three regions of the cornea under the influence of the Klotho null mutation, when comparing with those of the wild types and the heterozygotes (Figure 8C–E). The accumulation of MDA was also increased, although without statistical significance (Figure 9C–E).

### 3.6. Klotho Null Mutation Leads to an Increase in Nrf2 Expression

**Figure 10:**
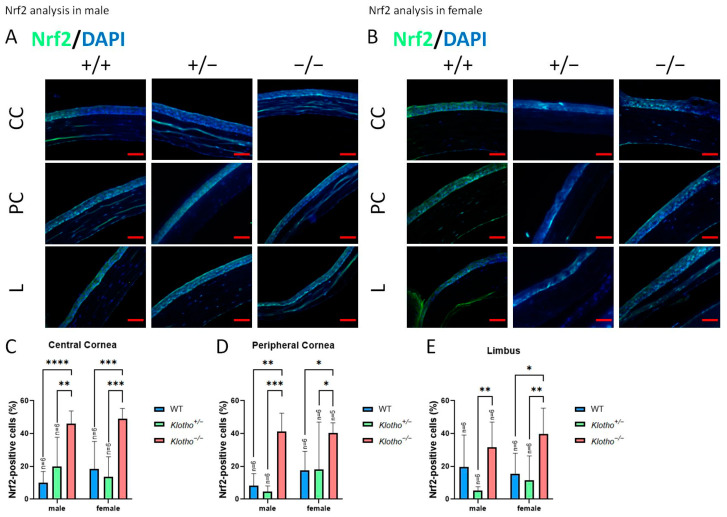
Representative photographs of corneal immunofluorescent detection with Nrf2 antibody and quantification analysis. (**A**) Nrf2 immunodetection in a male cornea. (**B**) Nrf2 immunodetection in a female cornea. (**C**–**E**) To analyze this quantitatively, Nrf2 expression is represented by the Nrf2-positive area/central cornea area, peripheral cornea area, and limbus area. Nrf2 expression is elevated in all cornea regions, irrespective of gender. Particularly, the increase in Nrf2 expression is highly significant in the central cornea (**C**). * *p* < 0.05, ** *p* < 0.01, *** *p* < 0.001, and **** *p* < 0.0001. The scale bars represent 50 μm.

Nrf2 was used as a marker for the expression of antioxidant genes. We also detected Nrf2 expression in the absence of the Klotho function (Figure 10A–E). The results show a general elevation in Nrf2 expression in all cornea regions, irrespective of gender. Particularly, the increase in Nrf2 expression is highly significant in the central cornea (Figure 10C).

### 3.7. Klotho Null Mutation Leads to a Comcimitant Increase in p53, Ki67, and Apoptosis

**Figure 11:**
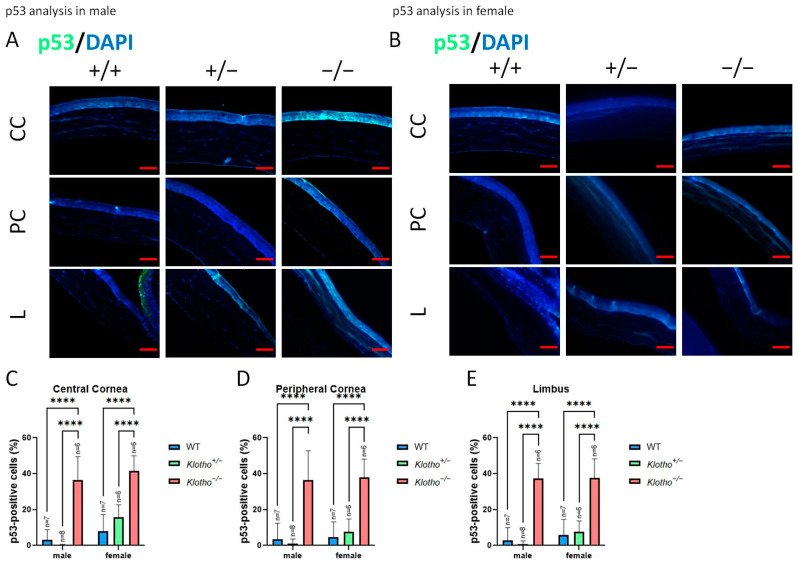
Representative photographs of corneal immunofluorescent detection with p53 antibody and quantification analysis. (**A**) p53 immunodetection in a male cornea. (**B**) p53 immunodetection in a female cornea. (**C**–**E**) To analyze this quantitatively, p53 expression is represented by the p53-positive area/central cornea area, peripheral cornea area, and limbus area. A general elevation in p53 expression is detected, irrespective of the cornea regions and gender. **** *p* < 0.0001. The scale bars represent 50 μm.

p53 was also detected in the absence of the Klotho function (Figure 11A–E). The results show a general elevation in p53 expression, irrespective of the cornea regions and gender.

**Figure 12:**
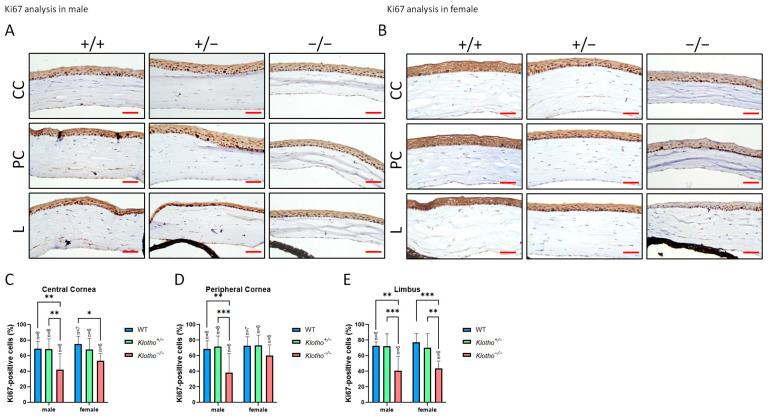
Representative photographs of corneal immunohistochemical detection with Ki67 antibody and quantification analysis. (**A**) Ki67 immunodetection in a male cornea. (**B**) Ki67 immunodetection in a female cornea. (**C**–**E**) To analyze this quantitatively, Ki67 expression is represented by the Ki67-positive area/central cornea area, peripheral cornea area, and limbus area. There is a general highly significant decrease in Ki67 expression, irrespective of the cornea regions and gender (**C**–**E**), except for the peripheral cornea where no significant increase occurs in the female mutants (**D**) * *p* < 0.05, ** *p* < 0.01, and *** *p* < 0.001. The scale bars represent 25 μm.

We also detected the expression of Ki67 in the absence of the Klotho function (Figure 12A–E). The results show a general highly significant decrease in Ki67 expression, irrespective of the cornea regions and gender (Figure 12C–E), except for the peripheral cornea where no significant increase was exhibited in the female mutants (Figure 12D).

**Figure 13:**
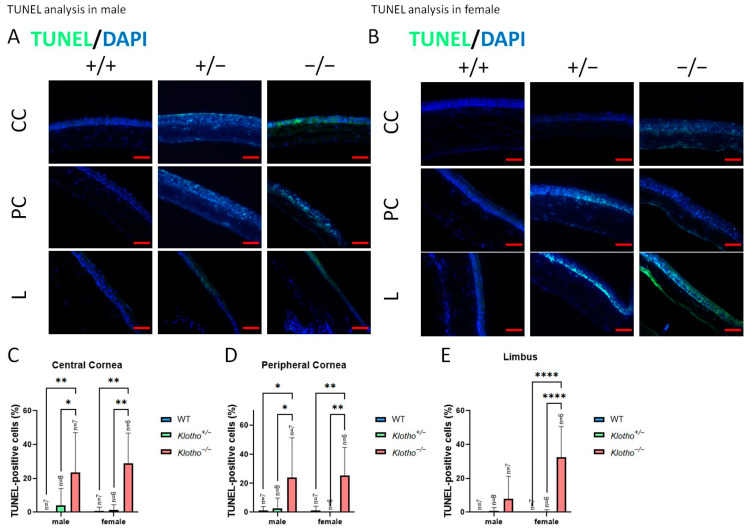
Representative photographs of corneal TUNEL and quantification analysis. (**A**) TUNEL in a male cornea. (**B**) TUNEL in a female cornea. (**C**–**E**) To analyze this quantitatively, TUNEL is represented by the TUNEL-positive area/central cornea area, peripheral cornea area, and limbus area. A general highly significant increase in apoptotic cells can be seen, irrespective of the cornea regions and gender (**C**–**E**), except for the limbus region of the cornea in the male mutants (**E**). * *p* < 0.05, ** *p* < 0.01, and **** *p* < 0.0001. The scale bars represent 50 μm.

We also detected apoptosis in the absence of the Klotho function (Figure 13A–E). The results show a general highly significant increase in apoptotic cells, irrespective of the cornea regions and gender (Figure 13C–E), except for the limbus region of the cornea in the male mutants (Figure 13E).

## 4. Discussion

Klotho has been extensively studied as an age-related gene that causes accelerated aging when it loses its function. The effects of the Klotho loss-of-function mutation have been studied in many organs, including the kidney, choroid plexus within the central nervous system, pituitary gland, brain, parathyroid gland, pancreas, ovary, testis, placenta, skeletal muscle, and many others [16,25,26]. The Klotho function has been found to be crucial almost everywhere in the body, and it is generally regarded as presenting antioxidant and anti-inflammatory activities. The common outcome of the consequence of the Klotho null mutation is therefore age-related pathogenesis, with the involvement of oxidative and inflammatory stresses [16,25,26].

Although the pathogenesis due to the Klotho null mutation has been extensively investigated in many organs, the effects on the ocular system remain largely unknown. Only a few reports on the retina [27,28,29], optic nerve [30], and lens [18], in relation to the Klotho null mutation, have been published, with the underlying pathogenesis mechanisms only specified in a limited manner. Adding to the previously published information, we show that the Klotho null mutation leads to lacrimal gland degeneration with characteristics of histopathological changes, altered neurosecretion, and reduced tear volume [31]. To date, there are no reports regarding the influence of the Klotho null mutation on the cornea.

Typical cornea aging traits in humans as well as in mice include corneal surface irregularities, a decrease in the epithelial barrier function, reduced corneal epithelial thickness, and increased thickness of the stroma [32,33,34,35]. These aging traits are known to cause age-related corneal epithelial diseases, most commonly expressed as dry eye complications that can affect vision. For example, a key age-related clinical feature of the cornea is the disruption of the epithelial barrier function, resulting in cornea surface irregularities [36]. Aged corneas also express some characteristics similar to those found in FECD [11]. Thus, the prevalence of DED and its associated visual impairment is widely known to increase significantly with age [32,36].

In the present study, we found an irregular topography on a corneal surface with the Klotho null mutation. Histological examinations revealed a reduced corneal epithelial cell density, endothelial cell-shedding, and decreased cornea stromal layer thickness in the absence of the Klotho function. Furthermore, guttae formation and the desquamation of wing cells were significantly increased in the Klotho loss-of-function mutant mice, comparable to the conditions of FECD and bullous keratopathy. These findings, to our knowledge, have not been reported to date.

Contrary to the common traits of cornea aging, the two interesting features observed specific for Klotho null mutant mice were (1) the unchanged epithelial layer thickness and (2) a thinner corneal stroma. The reduced stroma thickness is in accordance with the decreased tissue cellularity and collagen matrix as common manifestations of eye aging [37,38], but contrary to previous reports that claim that the cornea stroma thickens with aging. We speculate that this contradictory finding can be due to the alterations in EMT-related proteins with Klotho null mutations. During EMT, epithelial E-cadherin is suppressed, concomitant with an increase in N-cadherin expression in the mesenchyme, a phenomenon called “the cadherin switch” [39]. Elevated expressions of MMP-2 and MMP-9 are related to EMT [39,40,41]. We found that both MMP-2 and MMP-9 were increased with the Klotho null mutation, in agreement with the commonly known increased MMP expression and decreased ECM proteins with aging, presumably due to the lack of a response from fibroblasts to the fibroblast growth factor in aged corneas [38,42].

The process of EMT fits with the findings of the unchanged epithelial layer thickness along with the reduced epithelial cell density and the reduction in corneal stroma thickness observed in this study. It is likely that the Klotho mutation causes EMT through the upregulation of N-cadherin, MMP2, and MMP9 in the stroma. Also, notably, the previous literature showed elevated expressions of both MMP-2 and MMP-9 in dry eyes [43], and both MMP-2 and MMP-9 have been shown to preferentially degrade basement membrane components and are implicated in corneal epithelial wound healing [44,45]. MMP-2 and MMP-9 are also known to affect corneal epithelial growth [45]. In the present study, the results show that cornea epithelial thickness is maintained, although the total cornea epithelium is reduced. The maintenance of cornea epithelial thickness is likely to benefit from the EMT process under the influence of elevated MMP-2 and MMP-9 expressions.

CECs are crucial for maintaining relative corneal de-swelling through their pump and barrier functions, and corneal thickness maintenance is regarded as an indirect indicator of corneal epithelial regeneration [15]. CECs are slowly lost over time with aging, and functional compensation is performed by the remaining adjacent CECs [15]. With the development of FECD, the accelerated aging mechanisms lead to a loss of endothelial cells [10,12]. However, in the early stage, it is difficult to differentiate FECD pathogenesis from the corneal changes in normal aging [11]; despite this, FECD is a leading cause of corneal endothelial (CE) degeneration and can result in impaired visual acuity, particularly in women [9,12].

In the present study, we detected endothelial cell-shedding, wing cell desquamation, and guttae formation in the corneas of Klotho loss-of-function mutant mice. Guttae formation was reported to be associates with the upregulation of EMT and senescence markers [46]. The increased incidence of guttae formation in the Klotho null mutant mice was also concomitant with an increase in EMT markers. Also, increased apoptosis was observed with the Klotho null mutation. Notably, increased apoptosis is a common sign of FECD, and FECD can be a cause of bullous keratopathy. The surface epithelium can be compromised in bullous keratopathy by abnormal cellular and basement membrane adhesions and can be prone to increased cellular shedding [14]. Guttae formation is a typical trait of FECD, due to the abnormal re-modeling of the ECM under the continuous influence of EMT signaling [2,12]. Our results show that MMP-9 expression occurs within the guttae, reflecting a status of abnormal ECM re-modeling. In addition, our previous publication confirmed the dry eye status of the Klotho loss-of-function mutant mice [31]. In the tears of keratoconjunctivitis sicca (KCS) patients, MMP-9 concentration and activity were increased, which lead to the destruction of cornea epithelial tight junctions, resulting in epithelial exfoliation with subsequence effects on the underlying wing cells [36,47]. Together, all these phenomena were detected in the Klotho null mutant mice, suggesting a common pathogenesis process.

Arguably, it has been reported that Klotho can inhibit the Wnt signaling pathway [39], and Wnt-1 are known as a corneal epithelial stem cell markers [15,48]. Upon corneal endothelial injuries, Wnt-1 is proven to maintain the endothelium and inhibit EMT through its downstream Wnt/β-catenin activation and the subsequent regulation of MMPs [8,41]. Our present results, however, do not show a significant increase in Wnt-1 expression with the Klotho null mutation, although an increasing trend is detected. Due to this lack of significance, we then sought to explain the pathogenetic phenotype through the oxidative stress and inflammation status under the influence of the Klotho null mutation. However, the effects of the Klotho null mutation on Wnt signaling in the cornea remained to be further investigated.

Oxidative stress regulates the expression and activity of MMP-2 [49] and has been well-recognized as a contributing factor to age-related pathogenesis [50,51]. DNA damage is one of the hallmarks of aging, and 8-OHdG is widely used as a marker of DNA damage [50]. Another widely used marker is MDA, a product of lipid peroxidation. These two markers are related to pathogenesis involved under the influence of highly oxidative stress. In the present study, elevated 8-OHdG and MDA were found in the corneas with Klotho null mutations, indicating high oxidative stress in the absence of the Klotho function. This situation of high oxidative stress was also demonstrated by the elevation in NLRP3 expression in the cornea. High oxidative stress can trigger inflammation and subsequently activate epithelial cells to produce MMPs [52], leading to the aforementioned abnormalities. Also, NLRP3 is involved in ECM remodeling [52] that eventually triggers EMT [15,47,53]. In clinical studies, inflammation is reported to activate MMP secretion and induce cornea epithelial cell apoptosis, resulting in the destruction of the cornea barrier and aggravating the dry eye status [54]. Another line of evidence to indicate the high oxidative stress with Klotho null mutations is the increased Nrf2 in the cornea, which is demonstrated in the present study. Nrf2 is a transcription factor that has been known to regulate oxidative stress, immune response, and inflammatory reaction, as well as tissue remodeling and fibrosis in many tissues [50,51,55,56]. The fact that Nrf-2 was elevated represented a higher demand for increased antioxidant protection [50]. This higher demand and the role of Nrf-2 signaling are demonstrated in various organs. For example, the impairment of the Nrf-2 pathway was shown to play a role in heart aging with a Klotho deficiency [57]. Another example is the effect of Klotho on ameliorating diabetic nephropathy, which was carried out through Nrf2 signaling activation in podocytes [58]. Also, the attenuation of age-related renal phenotypes due to α-klotho deficiency was shown to be mediated by the Nrf-2 signaling pathway [59]. It is likely that Klotho loss-of-function mutant mice cause a compensatory activation of the Nrf-2 pathway in the cornea, but this activation did not ameliorate the pathological features found in the present study. Whether a blockage of the downstream factors subsequent to Nrf-2 activation occurs in the absence of the Klotho function or other alternative pathways are involved in the cornea pathological features found in the present study remains to be explored. Also, arguably, a Klotho expression was not detected in the wild-type corneas at 8 weeks of age (Supplementary Figure S2). The pathological features observed in this study are therefore more likely due to a loss of the Klotho function in the systemic circulation.

To further characterize the effects of high oxidative stress and inflammation on corneas with Klotho null mutations, we demonstrated the reduction in Ki-67 with a concomitant increase in p53 and elevated apoptotic activities in the cornea epithelium. Ki-67 is a marker of actively cycling cells and has been shown to decrease with aging [32,60,61], while p53 is an apoptosis marker [62] and has been shown to increase in human conjunctiva cells with aging [32]. With a normal Klotho function, p53 is inhibited [62]. In the absence of the Klotho function, it is likely that DNA damage in cornea epithelial cells, as demonstrated by the increased 8-OHdG expression, trigger the reduction in Ki-67, and a continuous Ki-67 reduction would lead to EMT and aging [12]. Likewise, DNA damage also leads to continuous p53 activation, resulting in cell apoptosis and aging [62,63,64].

## 5. Conclusions

The significance of the Klotho null mutant mouse model is that it can fulfill the lack of a unified cell or tissue culture system for corneal epithelial and endothelial cells, which limits the study of senescence phenotypes in these cell types. Moreover, no animal model of cornea endothelial degeneration has been established to be comparable to human clinical conditions, for example, in the situation of FECD. The lack of an in vivo model has impeded the studies on FECD pathogenesis and the development of interventions to aid its progression. In light of this disadvantage, Klotho null mutant mice can fill the gap by providing a study model for FECD research.

## Figures and Tables

**Table 1:** Occurrence of guttae formation, wing cell desquamation, and endothelial cell-shedding in the three Klotho genotypes and for both genders.

	+/+		+/−		−/−	
	Male	Female	Male	Female	Male	Female
Wing cell desquamation	1/7 (14.3%)	1/7 (14.3%)	2/7 (28.6%)	0/6 (0%)	2/4 (50%)	3/5 (60%)
Endothelial cell-shedding	0/7 (0%)	0/7 (0%)	0/7 (0%)	0/6 (0%)	2/4 (50%)	3/5 (60%)
Guttae	0/7 (0%)	0/7 (0%)	1/7 (14.3%)	1/6 (16.7%)	3/4 (75%)	5/5 (100%)

## Data Availability

The data presented in this study are available from the corresponding author upon reasonable request.

## Supplementary Materials

The following supporting information can be downloaded at: https://www.mdpi.com/article/10.3390/biology13030133/s1, Figure S1: Absence of Klotho expression in the cornea as detected by immunohistochemistry. Figure S2: Illustration of cornea division into the central, peripheral, and limbal parts.
