# Supplementary material for: Absence of the Klotho Function Causes Cornea Degeneration with Specific Features Resembling Fuchs Endothelial Corneal Dystrophy and Bullous Keratopathy
Source: Biology (Basel). 2024 Feb 20;13(3):133. doi: 10.3390/biology13030133 (PMC10968125; doi:10.3390/biology13030133)
Supplement: Supplementary file 1 [file biology-13-00133-s001.zip › biology-2833508-supplementary.pdf]

Supplementary Figure S1

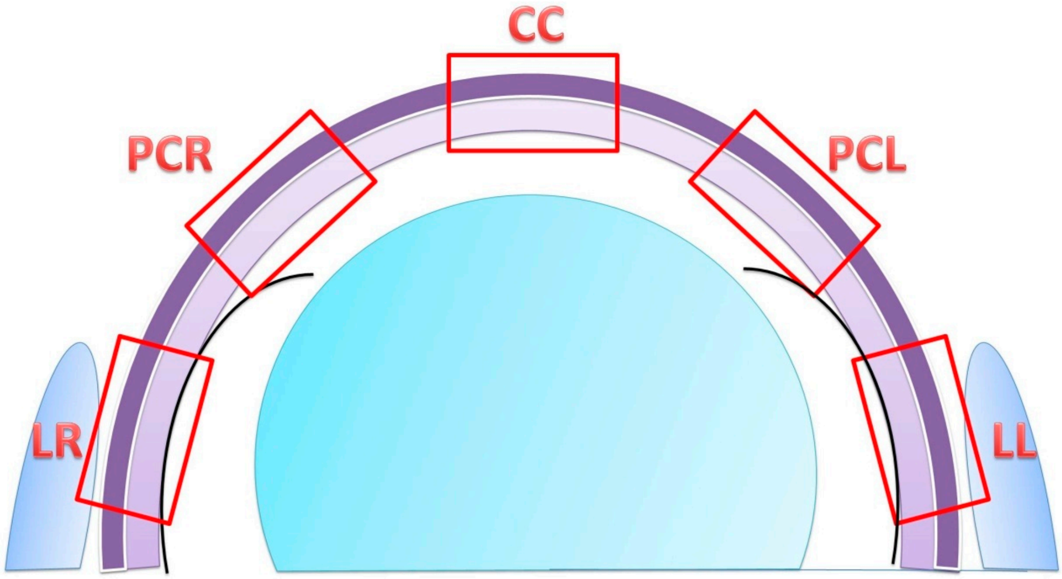

Figure S1 legend

Illustration of cornea division into the central, peripheral, and limbal parts

Quantification of all staining was allocated according to the central, peripheral, and limbal parts along the cornea. The central cornea (CC) was de-fined as within 200 um, right (R) or left (L), from the apex of the cornea. The limbus (L) was regarded to start at the base of the iris. The peripheral cornea (PC) was defined to locate within 200 um, right or left, from the middle point between the starting point of limbus and the apex of the cornea.

Supplementary Figure S2

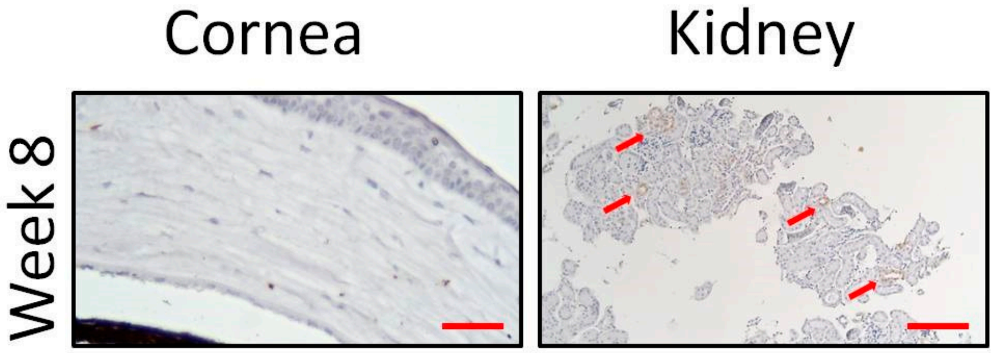

Figure S2 legend

Absence of Klotho expression in the cornea as detected by immunohistochemistry

Klotho expression was detected by immunohistochemistry. The results showed no expression in the wildtype cornea at 8 weeks of age. A parallel positive control detection was performed for detection of Klotho expression in the kidney. Evident expression (indicated by red arrows) was observed in the kidney from the same mouse. The scale bars represent 25μm.
